# Static platelet adhesion, flow cytometry and serum TXB_2 _levels for monitoring platelet inhibiting treatment with ASA and clopidogrel in coronary artery disease: a randomised cross-over study

**DOI:** 10.1186/1479-5876-7-42

**Published:** 2009-06-09

**Authors:** Andreas C Eriksson, Lena Jonasson, Tomas L Lindahl, Bo Hedbäck, Per A Whiss

**Affiliations:** 1 Division of Drug Research/Pharmacology, Department of Medical and Health Sciences, Linköping University, SE-581 85 Linköping, Sweden; 2 Division of Cardiology, Department of Medical and Health Sciences, Linköping University, SE-581 85 Linköping, Sweden; 3 Department of Clinical Chemistry, Laboratory Medicine, University Hospital, SE-581 85 Linköping, Sweden

## Abstract

**Background:**

Despite the use of anti-platelet agents such as acetylsalicylic acid (ASA) and clopidogrel in coronary heart disease, some patients continue to suffer from atherothrombosis. This has stimulated development of platelet function assays to monitor treatment effects. However, it is still not recommended to change treatment based on results from platelet function assays. This study aimed to evaluate the capacity of a static platelet adhesion assay to detect platelet inhibiting effects of ASA and clopidogrel. The adhesion assay measures several aspects of platelet adhesion simultaneously, which increases the probability of finding conditions sensitive for anti-platelet treatment.

**Methods:**

With a randomised cross-over design we evaluated the anti-platelet effects of ASA combined with clopidogrel as well as monotherapy with either drug alone in 29 patients with a recent acute coronary syndrome. Also, 29 matched healthy controls were included to evaluate intra-individual variability over time. Platelet function was measured by flow cytometry, serum thromboxane B_2 _(TXB_2_)-levels and by static platelet adhesion to different protein surfaces. The results were subjected to Principal Component Analysis followed by ANOVA, t-tests and linear regression analysis.

**Results:**

The majority of platelet adhesion measures were reproducible in controls over time denoting that the assay can monitor platelet activity. Adenosine 5'-diphosphate (ADP)-induced platelet adhesion decreased significantly upon treatment with clopidogrel compared to ASA. Flow cytometric measurements showed the same pattern (r^2 ^= 0.49). In opposite, TXB_2_-levels decreased with ASA compared to clopidogrel. Serum TXB_2 _and ADP-induced platelet activation could both be regarded as direct measures of the pharmacodynamic effects of ASA and clopidogrel respectively. Indirect pharmacodynamic measures such as adhesion to albumin induced by various soluble activators as well as SFLLRN-induced activation measured by flow cytometry were lower for clopidogrel compared to ASA. Furthermore, adhesion to collagen was lower for ASA and clopidogrel combined compared with either drug alone.

**Conclusion:**

The indirect pharmacodynamic measures of the effects of ASA and clopidogrel might be used together with ADP-induced activation and serum TXB_2 _for evaluation of anti-platelet treatment. This should be further evaluated in future clinical studies where screening opportunities with the adhesion assay will be optimised towards increased sensitivity to anti-platelet treatment.

## Background

Anti-platelet drugs such as acetylsalicylic acid (ASA) and clopidogrel are routinely used to prevent thrombosis in cardiovascular disease. The benefits of ASA have been clearly demonstrated by the Anti-platelet Trialists' Collaboration [1]. They found that ASA therapy reduces the risk by 25% of myocardial infarction, stroke or vascular death in "high-risk" patients. When using the same outcomes as the Anti-platelet Trialists' Collaboration on a comparable set of "high-risk" patients, the CAPRIE-study showed a slight benefit of clopidogrel over ASA [2]. Furthermore, the combination of clopidogrel and ASA has been shown to be more effective than ASA alone for preventing vascular events in patients with unstable angina [3] and myocardial infarction [4,5] as well as in patients undergoing percutaneous coronary intervention (PCI) [6,7]. Despite the obvious benefits from anti-platelet therapy in coronary disease, low response to clopidogrel has been described by several investigators [8-10]. A lot of attention has also been drawn towards low response to ASA, often called "ASA resistance". The concept of ASA resistance is complicated for several reasons. First of all, different studies have defined ASA resistance in different ways. In its broadest sense, ASA resistance can be defined either as the inability of ASA to inhibit platelets in one or more platelet function tests (laboratory resistance) or as the inability of ASA to prevent recurrent thrombosis (i.e. treatment failure, here denoted clinical resistance) [11-13]. The lack of a general definition of ASA resistance results in difficulties when trying to measure the prevalence of this phenomenon. Estimates of laboratory resistance range from approximately 5 to 60% depending on the assay used, the patients studied and the way of defining ASA resistance [11,13]. Likewise, lack of a standardized definition of low response to clopidogrel makes it difficult to estimate the prevalence of this phenomenon as well [8]. The principles of existing platelet assays, as well as their advantages and disadvantages, have been described elsewhere [14-18]. In short, assays potentially useful for monitoring treatment effects include those commonly used in research such as platelet aggregometry and flow cytometry as well as immunoassays for measuring metabolites of thromboxane A_2 _(TXA_2_). Also, the PFA-100™, Multiplate™ and the VerifyNow™ are examples of instruments commercially developed for evaluation of anti-platelet therapy. However, no studies have investigated the usefulness of altering treatment based on laboratory findings of ASA resistance [19]. Regarding clopidogrel, there are recent studies showing that adjustment of clopidogrel loading doses according to vasodilator-stimulated phosphoprotein phosphorylation index measured utilising flow cytometry decrease major adverse cardiovascular events in patients with clopidogrel resistance [20,21].

The current study used a randomised cross-over design in order to investigate the effects on platelets of dual therapy with ASA and clopidogrel as well as the effects of either drug alone in patients with a recent acute coronary syndrome. Platelet function was assessed by means of flow cytometry, serum TXB_2_-levels and by measuring static platelet adhesion to proteins in microplates. The aim was to evaluate the usefulness of the static platelet adhesion assay for measuring the effects of ASA and clopidogrel. Static adhesion is an aspect of platelet function that has not been investigated in earlier studies of the effects of platelet inhibiting drugs. Consequently, static platelet adhesion is not measured by any of the current candidate assays for clinical evaluation of platelet function. The static platelet adhesion assay offers an opportunity for simultaneous measurements of the combined effects of several different platelet activators on platelet function. In this study, platelet adhesion to albumin, collagen and fibrinogen was investigated in the presence of soluble platelet activators including adenosine 5'-diphosphate (ADP), adrenaline, lysophosphatidic acid (LPA) and ristocetin. Collagen, fibrinogen, ADP and adrenaline are physiological agents that are well-known for their interactions with platelets. Ristocetin is a compound derived from bacteria that facilitates the interaction between von Willebrand factor (vWf) and glycoprotein (GP)-Ib-IX-V on platelets, which otherwise occurs only at flow conditions [22]. The static nature of the assay therefore prompted us to include ristocetin in order to get a rough estimate on GPIb-IX-V dependent events [23]. LPA is a phospholipid that is produced and released by activated platelets and that also can be generated through mild oxidation of LDL [24]. It was included in the present study since it is present in atherosclerotic vessels and suggested to be important for platelet activation after plaque rupture. Finally, albumin was included as a surface since the platelet activating effect of LPA can be detected when measuring adhesion to such a surface [25]. Thus, by the use of different platelet activators, several measures of platelet adhesion were obtained simultaneously. This means that the possibilities to screen for conditions potentially important for detecting effects of platelet-inhibiting drugs far exceeds the screening abilities of other platelet function tests. Consequently, the static platelet adhesion assay is very well suited for development into a clinically useful device for monitoring platelet inhibiting treatment. Also, it has earlier been proposed that investigating the combined effects of two activators on platelet activity might be necessary in order to detect effects of ASA and other antiplatelet agents [26]. This is a criterion that can easily be met by the static platelet adhesion assay. Through the screening procedure we found different conditions where the static adhesion was influenced by the drug given. This suggests that the assay is able to detect treatment effects, but further studies are needed in order to refine the measurements.

## Methods

### Study design

**Figure 1:**
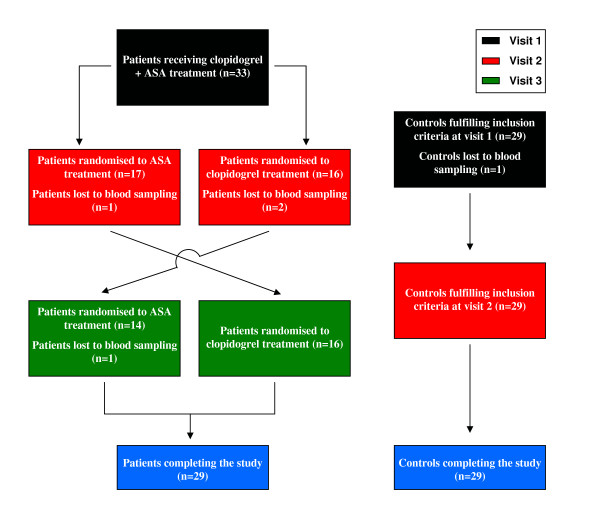
**Flow chart showing the inclusion of patients and controls**. Patients and controls were included consecutively. Blood samples from controls were drawn at two different occasions separated by 2–5.5 months. All patients entering the study received ASA combined with clopidogrel and blood sampling was performed 1.5–6.5 months after initiating the treatment. This was followed by a randomised cross-over enabling all patients to receive monotherapy with both ASA and clopidogrel. The patients received monotherapy for at least 3 weeks and for a maximum of 4.5 months before performing blood sampling. A total of 33 patients and 30 controls entered the study. In the end, 29 patients and 29 controls completed the study.

The study was approved by the Research Ethics Committee of Linköping University, Linköping, Sweden and the Medical Product Agency, Sweden (EudraCT Number 2005-003927-38). A total of 33 patients recently diagnosed with acute coronary syndrome were included on a consecutive basis from the Department of Cardiology at the University Hospital in Linköping, Sweden (Figure 1). Exclusion criteria were type 1 diabetes, immunologic or malignant disease, hepatic or kidney disease, heart failure NYHA class III-IV, heart valve disease, thoracal epidural anaesthesia or treatment with antibiotics, immunosuppressive drugs or continuous use of non-steroidal anti-inflammatory drugs (NSAID). At the index event, 8 patients received a bare metal stent and 15 received a drug-eluting stent following coronary angioplasty. During the course of the study, two patients were lost because of recurrent myocardial infarction and two left the study by their own decisions. Thus 29 patients, 19 males and 10 females, completed the study. When entering the study the male patients were on average 57 years old (range 40–69 years), while mean age for the female patients were 60 years (range 52–66 years). In parallel we collected samples from 30 healthy controls matched for age and gender. Only blood from controls declaring that they had not used any anti-platelet medication for two weeks prior to the study was used. For every control, samples were taken at two occasions separated by 2–5.5 months (Figure 1). One of the controls was excluded because of intake of NSAIDs meaning that a total of 29 controls, 19 males and 10 females, completed the study. At study entry the mean age of the male controls were 59 years (range 40–69 years), while mean for the female controls were 60 years (range 51–65 years).

Blood was drawn from patients at three different occasions (Figure 1). The first sample was drawn after all patients had received combined treatment with ASA (75 mg/day) and clopidogrel (75 mg/day) for 1.5–6.5 months after the index event. The study then used a randomised cross-over design meaning that half of the patients received ASA as monotherapy while half received only clopidogrel (75 mg/day for both monotherapies). The monotherapy was then switched for every patient so that all patients in total received all three therapies. Samples for evaluation of the monotherapies were drawn after therapy for at least 3 weeks and at the most for 4.5 months. Most of the differences in treatment length can be ascribed to the fact that the national recommendations for treatment in this patient group were changed during the course of the study. The allocation to monotherapy was blinded for the laboratory personnel. In general, the use of three different treatments for intra-individual comparisons in a cross-over design is different from previous studies on ASA and clopidogrel, which have mainly been concerned with only two treatment alternatives.

Whole blood was drawn from antecubital veins and collected in (1) tubes containing sodium heparin (final conc. 17 units/mL) for platelet adhesion analysis, (2) tubes with no additives for measurements of serum TXB_2_ and (3) tubes containing sodium citrate (final conc. 0.129 mol/L) for flow cytometric analysis (patients only). To obtain platelet rich plasma (PRP) for platelet adhesion analysis, 8 mL blood was transferred from sodium heparin tubes to a single plastic centrifuge tube. This single tube was then centrifuged for 20 min at 205 × g resulting in the production of a PRP supernatant. Blood obtained in serum tubes were allowed to clot at room temperature followed by centrifugation for 10 min at 1000 × g. The serum was transferred to eppendorf-tubes and stored at -70°C until analysis of TXB_2_. For patients, blood samples were also drawn into lithium heparin-tubes and K_2_EDTA-tubes for biochemical analysis at the accredited Department of Clinical Chemistry at the University Hospital in Linköping, Sweden. The lithium heparin-tubes were used for analysis of plasma concentrations of C-reactive protein (CRP), cholesterol, triglycerides, LDL-cholesterol, HDL-cholesterol, apolipoprotein-A1 (Apo-A1) and apolipoprotein-B (Apo-B), utilising the clinical chemistry analyzer Advia 1650 from Roche. Concentrations of platelets and leukocytes were determined from the K_2_EDTA-samples.

### Static platelet adhesion

Static platelet adhesion was measured as previously described [27]. Ninety-six well microplates (Nunc Maxisorp, Roskilde, Denmark) were coated with proteins by the addition of 100 μL/well of 2 mg/mL human albumin (Octapharma AB, Stockholm, Sweden), 0.1 mg/mL bovine collagen I (RnDsystems, Abingdon, UK) or 2 mg/mL human fibrinogen (American Diagnostica Inc., Greenwich, Connecticut, USA) followed by incubation at 4°C at least overnight and for a maximum of 7 days. The microplates were then washed two times in 0.9% NaCl by plate inversion followed by the addition of 25 μL 0.9% NaCl or 25 μL MgCl_2 _(5 mmol/L final concentration) and 25 μL of platelet activators. The soluble platelet activators were ADP and LPA from Sigma-Aldrich (St Louis, Missouri, USA), adrenaline from Merck NM AB (Stockholm, Sweden) and ristocetin from Diagnostica Stago (Asnières-sur-Seine, France) (Additional file 1: Variables). Experiments were performed both in the absence and presence of MgCl_2 _since MgCl_2 _has been shown to affect platelet adhesion to the protein surfaces tested in this study [27,28]. The microplates were left for 20 min and then 50 μL PRP diluted 4 times with 0.9% NaCl was added. Platelets were then allowed to attach to the surfaces for 1 h at room temperature without shaking. After incubation, unbound platelets were removed by washing twice in 0.9% NaCl by plate inversion and 140 μL of a sodium citrate/citric acid buffer (0.1 mol/L, pH 5.4) containing 0.1% Triton X-100 and 1 mg/mL *p*-nitrophenyl phosphate (Sigma-Aldrich) was added. Background absorbance was measured at 405 nm using a Spectramax microplate reader (Molecular Devices, Sunnyvale, California, USA) and the microplates were then incubated for 40 min at room temperature during shaking. In parallel, 50 μL PRP as well as 50 μL 0.9% NaCl were added to wells on a separate microplate. Both PRP and NaCl wells were treated with 140 μL of the sodium citrate/citric acid buffer described above followed by background absorbance measurements and consequently served as controls for 100% and 0% adhesion respectively. During the 40 min incubation, an enzymatic reaction occurred between added phosphatase substrate and platelet acid phosphatase. Adding 100 μL 2 mol/L NaOH to all wells (including 100% and 0%) stopped the reaction and resulted in a colour change of the developed product. Absorbance was measured at 405 nm with automatic reduction of background absorbance and percentage platelet adhesion was calculated.

### Flow cytometry

Platelet expression of P-selectin and binding of fibrinogen were measured by flow cytometry as indicators of platelet activation [29-32]. To tubes intended for fibrinogen binding analysis, 10 μL FITC-conjugated chicken anti-fibrinogen-antibodies (Diapensia, Linköping, Sweden) was mixed with 100 μL Hepes buffer. Hepes buffer containing EDTA was mixed with 10 μL of the same antibody for estimation of background fluorescence. For P-selectin measurements, 10 μL FITC-conjugated chicken anti-P-selectin-antibodies (Diapensia) were added to 100 μL Hepes buffer. Samples containing 10 μL anti-insulin-FITC (Diapensia) and 100 μL Hepes buffer served as indicators of background fluorescence. Whole blood (10 μL) was added to all tubes followed by addition of 10 μL ADP, the thrombin receptor PAR1 activating peptide SFLLRN (The Biotechnology Centre of Oslo, Oslo University, Norway) or vehicle (Hepes buffer) (Additional file 1: Variables). After incubation for 10 minutes, the reaction was stopped by addition of 1 mL Hepes buffer. Before flow cytometric analysis, samples were diluted three times in Hepes buffer and incubated for 30 min, while protected from light. Flow cytometric analysis was performed with the instrument Beckman Coulter Epics XL-MCL (Beckman Coulter Inc., Fullerton, California, USA) with computer software program (Expo 32 ADC, Beckman Coulter Inc.). The fluorescence intensity was checked daily with fluorescent beads (Flow set, Beckman Coulter Inc.). 5000 events were collected based on their forward and side scatter properties.

### TXB_2 _Enzyme Immuno Assay

Serum levels of TXB_2 _were measured with a commercial enzyme immuno assay (EIA) kit according to the manufacturers' instructions (Cayman Chemical, Ann Arbor, Michigan, USA). Amount of TXB_2 _present in serum was calculated with the use of a data analysis tool developed by Cayman Chemical [33].

### Statistics

The variables measured were subjected to Principal Component Analysis (PCA) with direct obliminal rotation using SPSS 14.0 software (SPSS Inc., Chicago, Illinois, USA). This technique analyses to what extent different variables are measuring the same concept and allows correlating variables to be ordered into separate factors [34]. The PCA performed in this study included a total of 69 variables. Each variable were included in the PCA as a composite of the results obtained from all data available for the specific variable. Thus, variables measured in both patients and controls (platelet adhesion and serum TXB_2_-levels) consisted of data from three measurements on patients and two on controls. All other variables were only analysed on patients, which resulted in three measurements that were included in the PCA. A variable was considered to be part of a factor when its loading was ≥ 0.4. After finding distinct factors, the composite variables included in the PCA were standardised according to Z-scores. This procedure transforms all variables to the same scale having a mean value of 0 and a standard deviation of 1. For each individual, a mean was calculated from the Z-scores of the variables that were found to belong to the same factor. From the Z-mean of the individuals, a Z-mean of the whole factor was calculated and further used for statistical comparisons of means. The factors, as well as some representative variables, were then analysed for treatment effects and for intra-individual variations within controls by Repeated Measures ANOVA. Differences between controls and patients were analysed by One-sample t-test. Correlations between factors were investigated with linear regression.

## Results

### Principal Component Analysis

In total the PCA grouped the initial 69 variables of platelet activation and routine clinical chemistry analyses into 15 different factors that we renamed according to the aspects they measured (Additional file 2: Factors). These names and/or the factor numbers are used throughout the article when describing and discussing the results of the present study. This procedure including screening followed by statistical complexity reduction is unusual for this type of study. Among the variables measuring platelet function, platelet adhesion was represented by eight factors, flow cytometry by two factors and serum TXB_2 _formed a separate factor. Visual inspection of the data of the healthy controls for the initial factor solution revealed possibilities for making the factors corresponding to platelet adhesion even simpler. Attention was paid at (1) different concentrations of the same soluble agonist on a specified surface, (2) the effects of weak agonists compared to basal adhesion and (3) the effect of an agonist compared to its combination with another agonist.

The first scenario was found in factor 1. Since all surfaces are represented with ADP at 1 and 10 μmol/L, it might be possible that addition of 1 μmol/L ADP results in maximal platelet adhesion with 10 μmol/L not contributing any further. In such a case it would be unnecessary to include the high concentration of ADP since it would not contribute any additional information. This was analysed by paired analysis for the two doses of ADP on every single surface. On all surfaces, ADP at 10 μmol/L was significantly different from 1 μmol/L ADP and all variables in Factor 1 were therefore kept on this basis. However, four of the variables in Factor 1 were excluded for other reasons (see next section).

The second scenario regarding the effect of weak agonists can be exemplified by Factor 5. It is possible that weak agonists do not increase platelet adhesion significantly compared to adhesion to the surface alone. As was the case for different doses of ADP, the weak agonist will then not contribute any relevant information regarding adhesion and could therefore be excluded. For Factor 5, adrenaline at 1 μmol/L was the only agonist that induced significantly increased adhesion compared to the surface alone and all others were consequently excluded from this factor. As for Factor 1, other reasons motivated the exclusion of adrenaline at 1 μmol/L as well from Factor 5 (see next section).

A special case was observed for Factor 8. Pairwise analysis of the data regarding adhesion to collagen in the presence of Mg^2+ ^showed that both adrenaline and LPA induced a weak albeit significant decrease in platelet adhesion. Since both LPA and adrenaline are platelet agonists, the decreased adhesion observed was considered irrelevant in this case and the variables were excluded.

Factor 4, 6 and 7 belongs to the third scenario in which comparisons were made between single agonist addition and addition with the same agonist in the same concentration combined with a second agonist. The combined addition was excluded unless it resulted in significantly increased adhesion compared to single agonist addition.

Finally, Factor 2 contained only variables that can be regarded as negative controls resulting in no platelet adhesion, as exemplified by albumin without any soluble activator. Such conditions can never detect inhibiting effects of drugs, which prompted us to exclude the whole factor.

### Intra-individual variation in healthy controls

**Figure 2:**
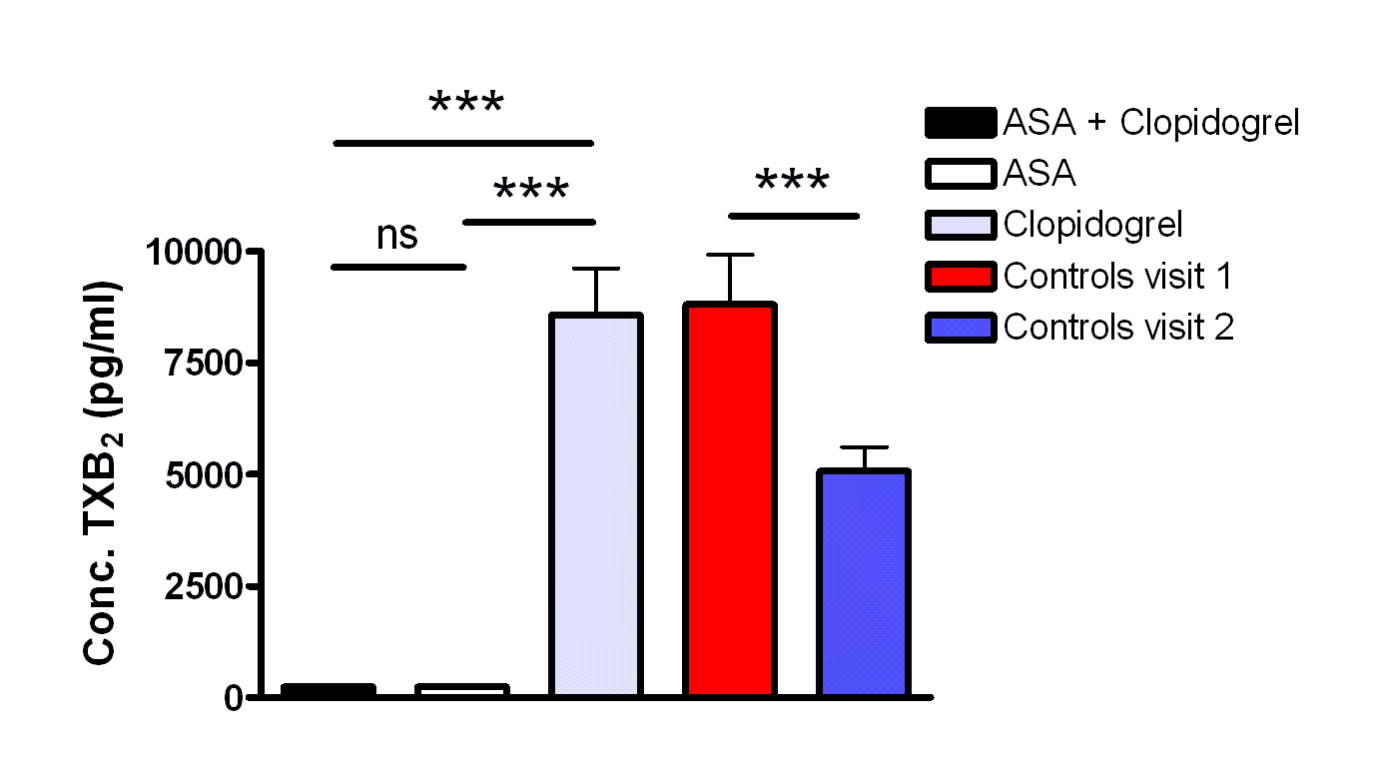
**Effect of platelet inhibiting treatment on serum TXB_2_-levels (Factor 13)**. Serum TXB_2_-levels (Factor 13) for patients (n = 29) and healthy controls (n = 29) are presented as mean + SEM. ASA alone or in combination with clopidogrel was significantly different from clopidogrel alone and compared to the mean of the controls (p < 0.001). Also, the difference between controls at visit 1 and visit 2 was significant. ***p < 0.001, ns = not significant.

Measurements of platelet adhesion and serum TXB_2_-levels were performed on healthy controls on two separate occasions (2–5.5 months interval) in order to investigate the presence of intraindividual variation in platelet reactivity and clotting-induced TXB_2_-production. The standardised Z-scores from the simplified factors were used for analysis by Repeated Measures ANOVA of the data from the healthy controls. We found significantly decreased platelet adhesion at the second compared to the first visit for ADP-induced adhesion (Factor 1, p = 0.012) and for adhesion to fibrinogen (Factor 5, p = 0.012). This intra-individual variability over time makes it difficult to draw any conclusions regarding effects of anti-platelet treatment. We therefore further analysed the individual variables constituting Factors 1 and 5 with Repeated Measures ANOVA in order to distinguish the variables that varied significantly over time. Variables being significantly different between visit 1 and visit 2 were then excluded and a new Repeated Measures ANOVA was performed on the new factors. After this modification, none of the factors corresponding to adhesion showed variation over time and these factors were then used for analysis on patients. Serum levels of TXB_2_, which constituted a separate factor, varied significantly in healthy controls at two separate occasions (Figure 2).

### Effects of platelet inhibiting treatment in coronary artery disease

**Figure 3:**
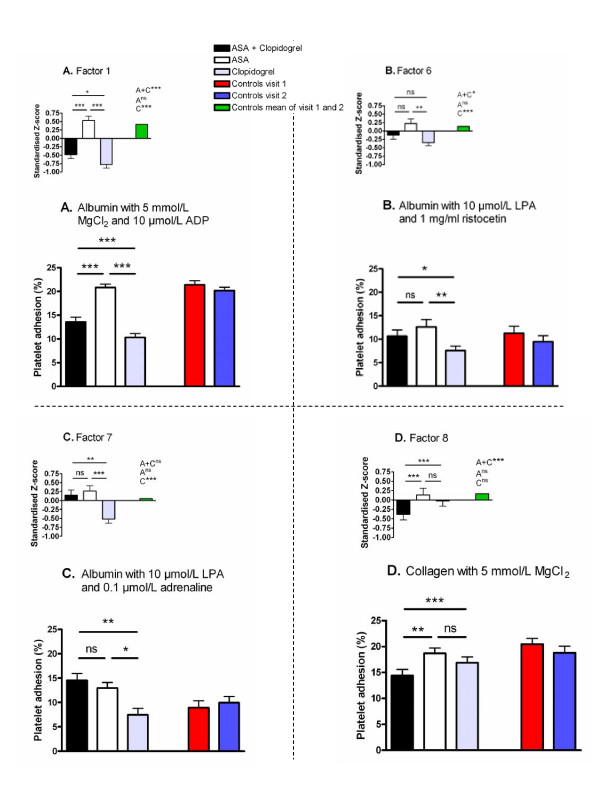
**The influence of ASA and clopidogrel on platelet adhesion**. The main figures are representative examples of the variables constituting the respective factors. The insets show the Z-scores for each factor. Also shown in the insets are the comparisons between the control means of visit 1 and 2 and treatment with ASA (A), clopidogrel (C) and the combination of ASA and clopidogrel (A+C). The respective figures show the effect of platelet inhibiting treatment on ADP-induced adhesion (Factor 1, Fig A), ristocetin-induced adhesion to albumin (Factor 6, Fig B), LPA-induced adhesion to albumin (Factor 7, Fig C) and adhesion to collagen (Factor 8, Fig D) for patients (n = 29) and healthy controls (n = 29). All values are presented as mean + SEM. *p ≤ 0.05, **p ≤ 0.01, ***p ≤ 0.001, ns = not significant.

**Figure 4:**
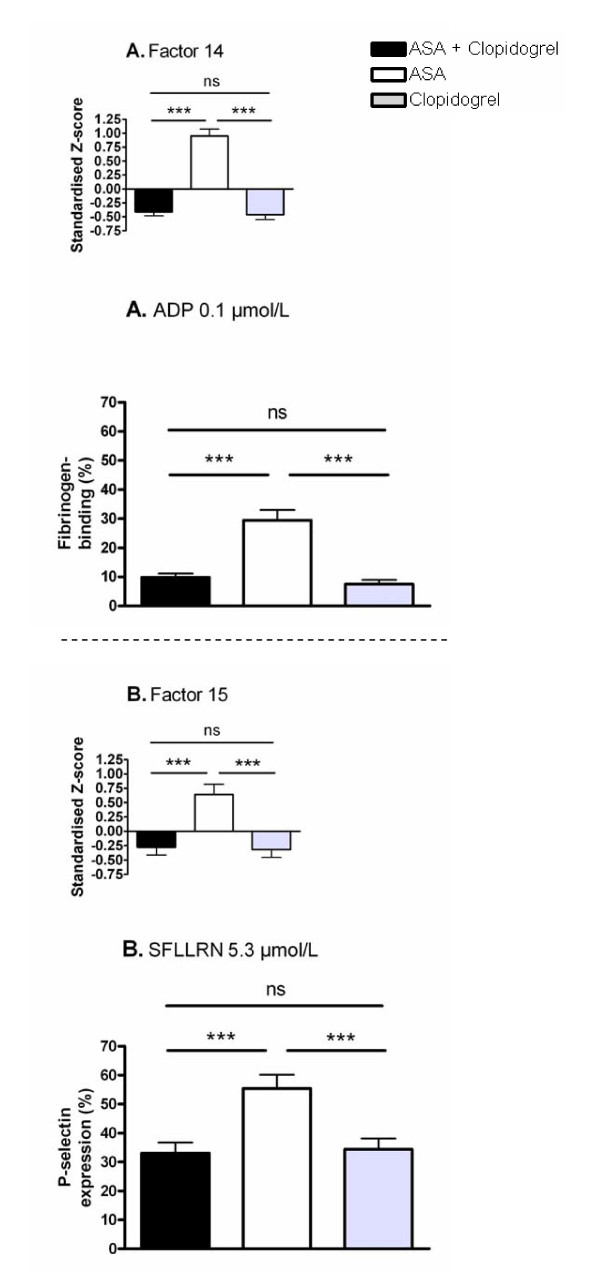
**The influence of ASA and clopidogrel on platelet activity measured by flow cytometry**. The effects of platelet inhibiting treatment on platelet activation detected by flow cytometry induced by ADP (Factor 14, Fig A) and SFLLRN (Factor 15, Fig B) on patients (n = 29). The main figures are representative examples of the variables constituting the respective factors. The insets show the Z-scores for each factor. All values are presented as mean + SEM. ***p < 0.001, ns = not significant.

When investigating possible effects of platelet-inhibiting treatment with Repeated Measures ANOVA, significant effects were seen for four of the factors corresponding to platelet adhesion. The factors that were not able to detect significant treatment effects were adrenaline-induced adhesion (Factor 3), ristocetin-induced adhesion (Factor 4) and adhesion to fibrinogen (Factor 5). Regarding adhesion factors detecting treatment effects, ADP-induced adhesion (Factor 1, Figure 3A inset) was significantly decreased by clopidogrel alone or by clopidogrel plus ASA compared with ASA alone. Surprisingly, platelet adhesion induced by ADP was lower for the monotherapy with clopidogrel compared to dual therapy. ADP-induced adhesion to albumin is shown as a representative example of the variables of Factor 1 (Figure 3A). Ristocetin-induced adhesion to albumin (Factor 6, Figure 3B inset) was significantly decreased by clopidogrel alone compared with ASA alone. This difference was also seen for ristocetin combined with LPA, which is shown as an example of a variable belonging to Factor 6 (Figure 3B). In Factor 7 (Figure 3C inset), corresponding to LPA-induced adhesion to albumin, we found clopidogrel to decrease adhesion compared with ASA and compared with ASA plus clopidogrel. These differences were reflected by the combined activation through LPA and adrenaline, which was a variable included in Factor 7 (Figure 3C). Finally, adhesion to collagen (Factor 8, Figure 3D) was significantly decreased by dual therapy compared with ASA alone or clopidogrel alone. As can be seen from the above description, monotherapy with clopidogrel resulted in significantly decreased adhesion compared to clopidogrel combined with ASA for Factors 1 and 7. This was also observed for the variable shown as a representative example of Factor 6 (Figure 3B). The two factors corresponding to flow cytometric measurements (Factors 14 and 15, Figure 4) both showed that ASA-treated platelets were more active than platelets treated with clopidogrel alone or clopidogrel plus ASA. Furthermore, serum TXB_2_-levels (Figure 2) was significantly decreased by ASA alone or by ASA plus clopidogrel compared with clopidogrel alone. Regarding the other measurements not directly measuring platelet function, significant differences were found for Factor 10 including HDL and for platelet count (Factor 12) but neither for the factor corresponding to inflammation (Factor 9) nor for Factor 11 including LDL. Factor 10 including HDL was found to be elevated by both ASA and clopidogrel monotherapies compared with dual therapy (p = 0.003 for ASA, p = 0.019 for clopidogrel, data not shown). Platelet count were found to be increased after dual therapy compared with both monotherapies (p < 0.001, data not shown).

### Comparisons between patients with coronary artery disease and controls

The factors were further analysed by One-sample t-test for differences between patients and controls. Thus, platelet adhesion and serum TXB_2_-levels of patients were compared to the mean of the two visits for controls included in the present study. ADP-induced platelet adhesion (Factor 1) and ristocetin-induced adhesion to albumin (Factor 6) were significantly decreased for patients treated with clopidogrel alone or in combination with ASA compared to healthy controls (Figure 3A–B). Monotherapy with clopidogrel resulted in significantly decreased platelet adhesion for LPA-induced adhesion to albumin (Factor 7) compared to controls (Figure 3C), while platelet adhesion to collagen (Factor 8) was significantly decreased for dual treatment (Figure 3D). Furthermore, adrenaline-induced adhesion (Factor 3) and ristocetin-induced adhesion (Factor 4) were increased for platelets on dual treatment compared to controls (p = 0.0002 and 0.0103 respectively, data not shown). Serum TXB_2_-levels were significantly decreased by dual therapy as well as by ASA alone compared to controls (Figure 2). For the flow cytometric measurements, patients were compared to historical reference values produced from healthy controls during routine clinical analysis. Consequently, we were not able to compare the factors established in this study corresponding to the flow cytometric measurements but instead compared the individual variables. After *in vitro *activation, binding of fibrinogen and expression of P-selectin were (with the exception of ADP-induced P-selectin expression on ASA-treated platelets) consistently decreased for patients compared to the reference values (Table 1). In opposite, basal levels of platelet activity were either equal, or slightly increased, for patients compared to controls (Table 1).

**Table 1:** Binding of fibrinogen and expression of P-selectin as measured by flow cytometry.

**Type of measurement**	**Activating agent**	**Reference values**	**ASA + Clopidogrel**	**ASA**	**Clopidogrel**
*Fibrinogen-binding*	Control	1 (0–3.4)	2.3 ± 0.3***	5.0 ± 2.5^ns^	2.4 ± 0.2***
	
	ADP 0.1	38 (17–59)	9.9 ± 1.3***	29.4 ± 3.6*	7.5 ± 1.4***
	
	ADP 0.6	74 (60–89)	32.5 ± 2.7***	62.1 ± 3.3**	22.9 ± 2.9***
	
	SFLLRN 5.3	76 (55–98)	28.8 ± 4.3***	48.5 ± 5.2***	20.2 ± 4.0***

*P-selectin expression*	Control	2 (0.9–3.1)	2.0 ± 0.2^ns^	4.8 ± 0.9**	4.3 ± 0.6***
	
	ADP 0.6	26 (10–42)	7.6 ± 0.8***	24.8 ± 2.4^ns^	10.9 ± 1.4***
	
	SFLLRN 5.3	88 (70–100)	33.0 ± 3.7***	55.4 ± 4.7***	34.4 ± 3.7***

Platelets from patients (n = 29) were activated *in vitro *with adenosine 5'-diphosphate (ADP; 0.1 and 0.6 μmol/L) or SFLLRN (5.3 μmol/L) followed by flow cytometric measurements of fibrinogen-binding or expression of P-selectin. Presented results are the mean-% of fibrinogen-binding and P-selectin expression ± SEM. Reference values (obtained earlier during routine analysis at the accredited Dept. of Clinical Chemistry at the University hospital in Linköping) are shown as mean with reference interval within parenthesis. Stars indicate significant differences for patients compared to reference values. *p ≤ 0.05, **p ≤ 0.01, ***p ≤ 0.001, ns = not significant.

### Linear regressions

**Figure 5:**
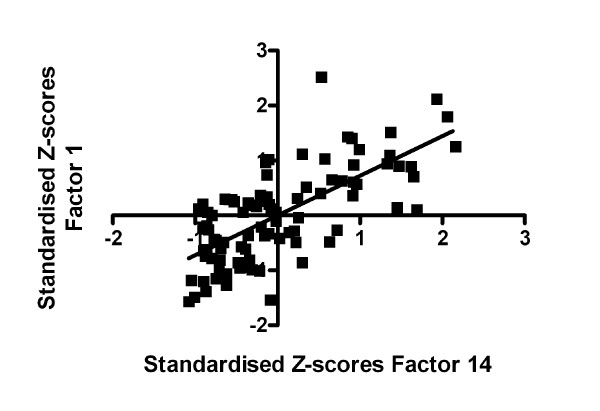
**Correlation between static platelet adhesion and flow cytometry**. Correlation between ADP-induced platelet adhesion (Factor 1) and ADP-induced platelet activation as measured by flow cytometry (Factor 14) for patients (n = 29) (r^2 ^= 0.49). Data included are from all three separate anti-platelet treatments (ASA and clopidogrel alone as well as ASA and clopidogrel combined).

Linear regression analyses were primarily focused on investigating possible correlations between any of the factors and (1) ADP-induced platelet adhesion and (2) serum TXB_2_-levels. These analyses were motivated since correlations with such pharmacodynamic measures of the effect of clopidogrel and ASA might indicate if a particular measure is dependent on ADP and/or TXB_2_. There was a connection between ADP-induced platelet adhesion and ADP-induced activation measured by flow cytometry (r^2 ^= 0.49, Figure 5). Other correlations with ADP-induced adhesion were observed for Factors 5–8 with r^2^-values ranging from 0.14–0.20. Furthermore, the two factors corresponding to platelet function measured by flow cytometry (Factors 14 and 15), correlated with an r^2^-value of 0.28. Regarding TXB_2_, regression analyses were only performed on samples with clopidogrel monotherapy since levels of TXB_2 _were totally suppressed when platelets were treated with ASA. However, serum TXB_2_-levels did not correlate with any of the other measurements.

## Discussion

With the aim of finding variables sensitive to clopidogrel and ASA-treatment, this study used a screening approach and measured several different variables simultaneously. To reduce the complexity of the material we performed PCA in order to find correlating variables that measured the same property. In this way the 54 measurements of platelet adhesion were reduced to 8 factors. Visual inspection revealed that each factor represented a separate entity of platelet adhesion and the factors could therefore be renamed according to the aspect they measured. We thus conclude that future studies must not involve all 54 adhesion variables, but instead, one variable from each factor should be enough to cover 8 different aspects of platelet adhesion. In addition to the adhesion data, the remaining 15 variables also formed distinct factors that were possible to rename according to measured property. It is notable that serum TXB_2 _formed a distinct group not correlated to any of the other measurements.

It is important that laboratory assays used for clinical purposes are reproducible and that they measure parameters that are not confounded by other variables. Some of the measurements performed in this study (clinical chemistry variables and platelet function measured by flow cytometry) are used for clinical analysis at accredited laboratories at the University hospital in Linköping. However, the reproducibility of the platelet adhesion assay was mostly unknown before this study [35]. Our initial results suggested that the factors corresponding to ADP-induced adhesion and adhesion to fibrinogen were not reproducible. We therefore excluded the most varied variables constituting these factors, which resulted in no intra-individual effects for healthy controls in the platelet adhesion assay. From this we conclude that many, but not all, measures of platelet adhesion are reproducible. Moreover, the static condition might limit the possibilities for translating the results from the adhesion assay into *in vivo *platelet adhesion occurring during flow conditions. However, platelet adhesion to collagen and fibrinogen is dependent on α_2_β_1_- and α_IIb_β_3_-receptors respectively in the current assay [23]. This suggests that the static platelet adhesion assay can measure important aspects of platelet function despite its simplicity. Furthermore, vWf dependent adhesion is not directly covered in the present assay although ristocetin-induced adhesion appears to be dependent on GPIb-IX-V and vWf [23].

We found that platelet adhesion to albumin, collagen and fibrinogen induced by ADP was suppressed during treatment with clopidogrel alone or in combination with ASA compared to treatment with ASA alone. The same pattern was also seen for the flow cytometric measures of ADP-induced activation. Furthermore, clopidogrel treatment suppressed both ADP-induced adhesion and ADP-induced activity measured by flow cytometry below the levels observed for healthy controls. These results are clear indications that both assays are able to detect the direct effect of inhibition of ADP-signalling by clopidogrel. It is also interesting to note that, upon ADP-challenge, the different assays correlated relatively well to each other. The variation between those factors not explained by the other can probably be ascribed to the different aspects of platelet function that the two assays measure. Flow cytometry measures the expression of activation-dependent receptors when platelets are in solution, while the adhesion assay measures the ability of platelets to adhere to a surface [27].

Measurements of serum TXB_2_-levels in healthy controls revealed significant intra-individual differences. This effect may be attributed to varying stress levels for the controls at the separate occasions since urinary levels of the TXA_2 _metabolite 2,3-dinor-TXB_2 _has been shown to correlate with urinary cortisol [36]. Also, urinary TXB_2 _follows a seasonal variation pattern [37]. According to our discussion above, this variation complicates the use of this measure of platelet activity in clinical routine. However, we also found that serum TXB_2_-levels were completely suppressed after ASA treatment compared to both clopidogrel treatment and controls. Consequently, despite the intra-individual variations, this shows that serum TXB_2 _is a good indicator of the ability of ASA to inhibit the cyclooxygenase pathway.

Some adhesion measures showed decreased adhesion by clopidogrel alone compared with clopidogrel plus ASA. Since dual therapy was always the initiating treatment, this difference may reflect the presence of more active platelets in the time frame closest to the index event and coronary revascularization. It has been proposed that reendothelialization after insertion of a bare metal stent is complete after approximately 3 months [38] and that arterial healing is even slower for a drug-eluting stent [39]. Consequently, it is possible that absence of endothelium after stenting contributes to the high initial platelet activity found in this study. A sustained inflammatory response after stenting may also partly explain the decreased levels of the negative acute phase reactant HDL cholesterol [40] during dual therapy compared with the monotherapies. Similarly, the sustained inflammatory response may explain the significant increase in platelet count during combination therapy. Platelet counts may initially decrease after surgical procedures followed by recovery and increased platelet count within the following period of time [41,42]. This would be represented by the dual therapy group in the present study. The change in platelet count is however complicated since it has also been reported that patients with unstable angina had a decreased platelet count compared with stable angina patients and controls [43].

From this discussion it is evident that the adhesion assay as well as flow cytometry can measure effects of clopidogrel when using ADP as activating stimuli. It is also evident that serum-TXB_2 _levels measure the effects of ASA. However, these measures focus on the primary interaction between the drugs and the platelets, which could be problematic when trying to evaluate the complex *in vivo *treatment effect. It has previously been found that only 12 of 682 ASA-treated patients (≈ 2%) had residual TXB_2 _serum levels higher than 2 standard deviations from the population mean [44]. Measurements of the effect of arachidonic acid on platelet aggregometry have also led to the conclusion that ASA resistance is a very rare phenomenon [45]. Thus, our study supports these previous findings that assays measuring the pharmacodynamic activity of ASA (to inhibit the COX-enzyme) seldom recognizes patients as ASA-resistant. This suggests that the cause of ASA-resistance is not due to an inability of ASA to act as a COX-inhibitor. Explanations for ASA resistance are diverse and include e.g. patient non-compliance, interactions with other drugs, platelet polymorphisms and sustained COX-activity by other cells [12,13]. Several studies also propose that ASA-resistant platelets have increased platelet activation through signalling pathways not directly involving TXA_2 _[46-49]. In line with these studies it has been proposed that the presence of ASA resistance should be evaluated by combining measurements of TXB_2_-formation with platelet aggregation [50]. We further suggest that direct measurements of ADP and TXA_2_-effects (in our case ADP-induced activation measured by adhesion or flow cytometry and serum TXB_2_-levels) must be combined with measures that are only partly dependent on ADP and TXA_2 _respectively. For instance, an adhesion variable partly dependent on TXA_2 _might be able to detect ASA resistance caused by increased signalling through other activating pathways. Such a scenario would be characterized by serum TXB_2 _values showing normal COX-inhibition while platelet adhesion is increased. This study employed a screening procedure in order to find such indirect measures of the effects of ASA and clopidogrel. Our results show inhibiting effects of clopidogrel compared to ASA on adhesion to albumin in the presence of LPA or ristocetin. This was also observed for our flow cytometric measurements with SFLLRN as activator, which confirms that SFLLRN is able to induce release of granule contents in platelets [51,52]. SFLLRN- and ADP-induced platelet activation, as measured by flow cytometry, was moderately correlated to each other and adhesion induced by LPA as well as ristocetin showed weak correlations with ADP-induced adhesion. These results further confirm that these measures of platelet activity are partly dependent on ADP. We have earlier shown that adhesion to albumin induced by simultaneous stimulation by LPA and adrenaline (a variable belonging to the LPA-factor in the present study) can be inhibited by inhibition of ADP-signalling *in vitro *[25]. This strengthens our conclusion that the effect on LPA-induced adhesion observed for clopidogrel is caused by inhibition of ADP-signalling. Also, the presence of LPA in atherosclerotic plaques and its possible role in thrombus formation after plaque rupture [24] makes it especially interesting for the *in vivo *setting of myocardial infarction. The collagen surface is different from the other stimuli since dual therapy results in significantly depressed platelet adhesion compared to both monotherapies. This indicates that adhesion to collagen is dependent on both ADP and TXA_2 _and this measure was the only adhesion-related factor that showed potential for being partly dependent on TXA_2_. However, the significant effects observed between treatments were rather small. Nevertheless, it has earlier been shown that platelet activation induced by collagen is reduced by intake of ASA [26,53]. Regarding the flow cytometric measurements there were no indications for platelet activity to be decreased for dual therapy compared to monotherapy with clopidogrel. However, platelet activation as measured by flow cytometry was in general decreased for patients having monotherapy with ASA compared to healthy controls. This indicates that flow cytometry is also able to detect effects of ASA.

## Conclusion

In this study we employed different assays in order to evaluate platelet function in patients treated with different anti-platelet regimens. Among these assays, the platelet adhesion assay had a certain role since it had not been used before for this clinical purpose. Actually, there are no assays of static platelet adhesion that have been used in previous studies aimed at investigating treatment effects of platelet inhibiting drugs. Importantly, this study shows that the static platelet adhesion assay is reproducible over time. We also showed that the static platelet adhesion assay as well as flow cytometry detected the ability of clopidogrel to inhibit platelet activation induced by ADP. Our results further suggest that other measures of platelet adhesion and platelet activation measured by flow cytometry are indirectly dependent on secreted ADP or TXA_2_. One such measure is adhesion to a collagen surface, which should be more thoroughly investigated for its ability to detect effects of clopidogrel and ASA. Likewise, due to its connection to atherosclerosis and myocardial infarction, the LPA-induced effect should be further evaluated for its ability to detect effects of clopidogrel. In conclusion, the screening procedure undertaken in this study has revealed suggestions on which measures of platelet activity to combine in order to evaluate platelet function. Speculatively, the ADP-mediated effects in the present adhesion assay in combination with serum TXB_2_, may be combined with LPA and collagen-induced adhesion for an optimal monitoring of clopidogrel and ASA therapy.

## Competing interests

The authors declare that they have no competing interests.

## Authors' contributions

ACE carried out the analysis of static platelet adhesion and serum TXB_2_-levels and performed the statistical analyses. All authors participated in the design of the study, co-operated in the drafting of the manuscript and read and approved the final version of the manuscript.

## Supplementary Material

Additional file 1 **All variables measured in the study**. A table showing all the variables that were measured in the study. Click here for file

Additional file 2 **The final factors used for ANOVA analyses**. A table showing the factors used for ANOVA analyses. Click here for file
