# Supplementary material for: Static platelet adhesion, flow cytometry and serum TXB2 levels for monitoring platelet inhibiting treatment with ASA and clopidogrel in coronary artery disease: a randomised cross-over study
Source: J Transl Med. 2009 Jun 9;7:42. doi: 10.1186/1479-5876-7-42 (PMC2699331; doi:10.1186/1479-5876-7-42)
Supplement: Additional file 1 — All variables measured in the study. A table showing all the variables that were measured in the study. [file 1479-5876-7-42-S1.pdf]

Additional file 1. All variables measured in the study.

The upper part of the table shows the five different surfaces used for measuring platelet adhesion and all different additions of external activators made for each surface. All numerals indicate concentration in μmol/L except for ristocetin where the unit of concentration is mg/mL. Semicolons separate different concentrations of the same agonist. The lower part shows the different activating stimuli used for the flow cytometric measurements of fibrinogen-binding and P-selectin surface expression respectively. Also shown are the other measures not involving platelet adhesion or flow cytometry. Abbreviations are as follows: ADP = adenosine 5'-diphosphate, Adr = adrenaline, Risto = ristocetin, LPA = lysophosphatidic acid, CRP = C-reactive protein.

Adhesion				
**Albumin**	**Albumin + 5 mmol/L $MgCl_2$**	**Collagen**	**Collagen + 5 mmol/L $MgCl_2$**	**Fibrinogen + 5 mmol/L $MgCl_2$**
Solvent	Solvent	Solvent	Solvent	Solvent
ADP 0.1; 1; 10	ADP 1; 10	ADP 0.1; 1; 10	ADP 1; 10	ADP 1; 10
Risto 1	Risto 1	Risto 1	Risto 1	Risto 1
Adr 0.1; 1	Adr 0.1; 1	Adr 0.1; 1	Adr 0.1; 1	Adr 0.1; 1
LPA 1; 10	LPA 1; 10	LPA 1; 10	LPA 1; 10	LPA 1; 10
LPA 1+Adr 0.1		LPA 1+Adr 0.1		
LPA 10+Adr 0.1		LPA 10+Adr 0.1		
LPA 1+Risto 1		LPA 1+Risto 1		
LPA 10+Risto 1		LPA 10+Risto 1		
Adr 0.1+Risto 1		Adr 0.1+Risto 1		
ADP 0.1+Risto 1		ADP 0.1+Risto 1		

Flow Cytometry		Other Measures		
**Fibrinogen-binding**	**P-selectin expression**	TXB_2	LDL-cholesterol	Cholesterol
ADP 0.1; 0.6	ADP 0.6	CRP	Apo-B	Triglycerides
SFLLRN 5.3	SFLLRN 5.3	Leukocyte count	HDL-cholesterol	
		Platelet count	Apo-A1	
