# Supplementary material for: Static platelet adhesion, flow cytometry and serum TXB2 levels for monitoring platelet inhibiting treatment with ASA and clopidogrel in coronary artery disease: a randomised cross-over study
Source: J Transl Med. 2009 Jun 9;7:42. doi: 10.1186/1479-5876-7-42 (PMC2699331; doi:10.1186/1479-5876-7-42)
Supplement: Additional file 2 — The final factors used for ANOVA analyses. A table showing the factors used for ANOVA analyses. [file 1479-5876-7-42-S2.pdf]

Additional file 2. The final factors used for ANOVA analyses.
Words in italics are the factor names according to the property the factor measures. All numerals indicate concentration in μmol/L except for ristocetin where the unit of concentration is mg/mL. Semicolons separate different concentrations of the same agonist. Crossed out variables were initially part of the respective factors but later on excluded for different reasons (see text). Abbreviations are as follows: ADP = adenosine 5'-diphosphate, Adr = adrenaline, Risto = ristocetin, LPA = lysophosphatidic acid, CRP = C-reactive protein.

**Factor 1**<br>*(ADP-induced adhesion)*	**Factor 2**<br>*(Absent adhesion)*		**Factor 3**<br>*(Adrenaline-induced adhesion)*	**Factor 4**<br>*(Ristocetin-induced adhesion)*
Albumin Mg ADP 1; 10<br>Collagen ADP 1<br>Collagen Mg ADP 1; 10<br>Fibrinogen Mg ADP 1<br>~~Albumin ADP 1; 10~~<br>~~Collagen ADP 10~~<br>~~Fibrinogen Mg ADP 10~~	~~Albumin~~<br>~~Albumin ADP 0.1~~<br>~~Albumin Adr 0.1~~<br>~~Albumin LPA 1~~<br>~~Albumin LPA 1+Adr 0.1~~<br>~~Albumin Mg~~<br>~~Albumin Mg Adr 0.1~~	~~Albumin Mg LPA 1; 10~~<br>~~Collagen~~<br>~~Collagen ADP 0.1~~<br>~~Collagen Adr 0.1~~<br>~~Collagen LPA 1; 10~~<br>~~Collagen LPA 1+Adr 0.1~~<br>~~Collagen LPA 10+Adr 0.1~~	Albumin Adr 1<br>Albumin Adr 0.1+Risto 1<br>Albumin Mg Adr 1<br>Collagen Mg Adr 1	Collagen Risto<br>Collagen Adr 0.1+Risto 1<br>Collagen Mg Risto 1<br>Fibrinogen Mg Risto 1<br>~~Collagen ADP 0.1+Risto 1~~<br>~~Collagen LPA 1+Risto 1~~<br>~~Collagen LPA 10+Risto 1~~
**Factor 5**<br>*(Adhesion to Fibrinogen)*	**Factor 6** *(Ristocetin-induced adhesion to albumin)*	**Factor 7** *(LPA-induced adhesion to albumin)*	**Factor 8**<br>*(Adhesion to Collagen)*	**Factor 9**<br>*(Inflammation)*
Fibrinogen Mg<br>~~Fibrinogen Mg Adr 1~~<br>~~Fibrinogen Mg Adr 0.1~~<br>~~Fibrinogen Mg LPA 1; 10~~	Albumin Risto 1<br>Albumin ADP 0.1+Risto 1<br>Albumin LPA 10+Risto 1<br>Albumin Mg Risto 1<br>~~Albumin LPA 1+Risto 1~~	Albumin LPA 10<br>Albumin LPA 10+Adr 0.1	Collagen Mg<br>Collagen Adr 1<br>~~Collagen Mg Adr 0.1~~<br>~~Collagen Mg LPA 1; 10~~	CRP<br>Leukocyte count
**Factor 10**<br>*(HDL)*	**Factor 11**<br>*(LDL)*	**Factor 12**<br>*(Platelet count)*	**Factor 14** *(ADP, flow cytometry)*	**Factor 15** *(SFLLRN, flow cytometry)*
HDL-cholesterol<br>Apo-A1	LDL-cholesterol<br>Apo-B<br>Cholesterol<br>Triglycerides	Platelet count<br>**Factor 13** *(TXB_2)*<br>TXB_2	Fibrinogen ADP 0.1; 0.6<br>P-selectin ADP 0.6	Fibrinogen SFLLRN 5.3<br>P-selectin SFLLRN 5.3
